# Phase II Study of Nanoliposomal Irinotecan (Nal-IRI) with 5-Fluorouracil and Leucovorin in Refractory Advanced High-Grade Neuroendocrine Cancer of Gastroenteropancreatic (GEP) or Unknown Origin

**DOI:** 10.3390/cancers17020224

**Published:** 2025-01-12

**Authors:** Sarbajit Mukherjee, Harsha Pattnaik, Sahithi Sonti, Mrinalini Ramesh, Prantesh Jain, Robert A. Ramirez, Christos Fountzilas, Deepak Vadehra, Kristopher Attwood, Renuka Iyer

**Affiliations:** 1 Roswell Park Comprehensive Cancer Center, Buffalo, NY 14203, USA; harsha.pattnaik@roswellpark.org (H.P.); sahithi.sonti@gmail.com (S.S.); prantesh.jain@roswellpark.org (P.J.); christos.fountzilas@roswellpark.org (C.F.); deepak.vadehra@roswellpark.org (D.V.); attwood3@buffalo.edu (K.A.); renuka.iyer@roswellpark.org (R.I.); 2 Department of Internal Medicine, University at Buffalo, Buffalo, NY 14203, USA; mramesh3@buffalo.edu; 3 Vanderbilt-Ingram Cancer Center, Nashville, TN 37232, USA; robert.ramirez@vumc.org

**Keywords:** gastroenteropancreatic NET, nanoliposomal irinotecan, neuroendocrine carcinomas, UGT1A1*28

## Abstract

Background: Neuroendocrine carcinomas (NECs) are treated with a frontline platinum–etoposide combination with no standard second-line therapies. We explored a novel combination of nanoliposomal irinotecan (Nal-IRI), 5-fluorouracil (5-FU), and leucovorin (LV) in advanced refractory NECs and investigated the impact of UGT1A1*28 polymorphism on treatment outcomes and toxicity. Methods: We conducted an open-label, single-arm, multi-center Phase 2 trial in advanced NEC patients of gastroenteropancreatic (GEP) or unknown origin with progression or intolerance to first-line therapy. Eligible patients received nal-IRI 70 mg/m^2^ and leucovorin 400 mg/m^2^, followed by 5-FU 2400 mg/m^2^ biweekly till disease progression or unacceptable toxicity. The primary endpoint was the objective response rate (ORR). Secondary endpoints included progression-free survival (PFS), overall survival (OS), and toxicity. Next-generation sequencing (NGS) was performed on blood/tissue samples at baseline and during treatment. Results: Eleven patients were enrolled, with nine evaluable for the primary endpoint. Seven were male, the median age was 66.7 years, and the median Ki-67 was 90%. We observed partial response in one patient, stable disease in six patients, and progressive disease in two patients. The median OS was 9.4 months (95% CI 2.9–29.3), and the median PFS was 4.4 months (95% CI 1.7–6.7). The most common adverse events were diarrhea (45%), nausea (45%), vomiting (45%), and fatigue (45%). The most common genetic mutations on NGS were TP53 (88.9%), CHEK2 (88.9%), and APC (33.3%). Patients with CHEK2 and APC mutation had longer PFS (*p* = 0.005 and *p* = 0.013, respectively). UGT1A1*28 polymorphism was not associated with OS, PFS, or toxicity. Conclusion: Nal-IRI with 5-FU/LV is a safe and effective treatment for refractory high-grade NECs of GEP or unknown origin. Future studies should explore novel combinations with Nal-IRI in high-grade NECs both in frontline and refractory settings.

## Simple Summary

This Phase 2 trial investigated the combination of nanoliposomal irinotecan (Nal-IRI), 5-fluorouracil (5-FU), and leucovorin (LV) in patients with advanced, refractory neuroendocrine carcinomas (NECs) of gastroenteropancreatic (GEP) or unknown origin. Eleven patients were enrolled, with nine evaluable for response. The treatment showed a partial response in one patient, stable disease in six, and progressive disease in two. The median overall survival was 9.4 months, and progression-free survival was 4.4 months. Common side effects included diarrhea, nausea, vomiting, and fatigue. Genetic analysis revealed that mutations in TP53, CHEK2, and APC were common, with CHEK2 and APC mutations linked to longer progression-free survival. The study found no significant association between the UGT1A1*28 polymorphism and treatment outcomes or toxicity. Overall, Nal-IRI with 5-FU/LV was found to be a safe and promising treatment option for refractory high-grade NECs, warranting further investigation in future trials.

## 1. Introduction

The incidence of neuroendocrine tumors (NETs) has been steadily rising over the past few decades [1,2]. Poorly differentiated grade 3 tumors, with Ki-67 ranging from 20 to 100%, fall into the group of neuroendocrine carcinomas (NECs). Gastroenteropancreatic (GEP) NETs represent about 55–70% of all NETs. The incidence of GEP NECs showed a significant leap from 1.5/1,000,000 cases in 1973 to 4.6/1,000,000 cases in 2012 [3].

The current standard for systemic therapy is a platinum plus etoposide combination, but the duration of benefit is short, and survival is less than one year [4]. The combination of temozolomide and capecitabine (CapTem) has shown efficacy but has limited response rates in non-pancreatic NETs [5]. The Nordic NEC study evaluated survival in patients who received platinum- or temozolomide-based regimens first-line and reported 11–13 month-survival in both groups [6]. Based on these results, the Eastern Cooperative Oncology Group (ECOG)—ACRIN Cancer research group investigated the optimal first-line regimen, CapTem vs. platinum–etoposide, in a randomized trial (NCT02595424). This study demonstrated that CapTem was not superior to platinum–etoposide chemotherapy but had a more favorable toxicity profile [7].

In addition to chemotherapy regimens, immunotherapy has also been studied in NECs. The addition of atezolizumab to chemotherapy in small-cell lung cancers, which have similar pathology as NECs, resulted in significant improvement in overall survival (OS) and progression-free survival (PFS) [8], which was the basis for the ongoing clinical trial (NCT05058651). This trial is randomizing extrapulmonary metastatic NEC patients to receive standard chemotherapy with or without atezolizumab to investigate the efficacy of frontline immunotherapy in advanced or metastatic poorly differentiated extrapulmonary NECs [9].

Irinotecan alone or in combination has shown promising efficacy in patients with small-cell lung cancer [10]. Morizane et al. conducted a randomized clinical trial that showed comparable effectiveness between etoposide/cisplatin and irinotecan/cisplatin regimens in GEP NECs [11].

UDP-glucuronosyltransferase1A1 (UGT1A1) is involved in the metabolism of various chemotherapeutic agents, including irinotecan. UGT1A1*28 and UGT1A1*93 polymorphism subtypes are poor metabolizers of irinotecan and its metabolite SN-38, leading to excess accumulation, which precipitates severe toxicity and requires dose adjustments [12,13]. The encapsulation of irinotecan in a liposomal nanoparticle (nal-IRI) allows for a longer circulation time for irinotecan, leading to an increase in irinotecan and its major active metabolite, SN-38 levels, in the tumor [14,15,16]. The NAPOLI-1 trial, a study of nanoliposomal irinotecan plus 5-fluorouracil (5-FU) in advanced pancreatic adenocarcinoma, demonstrated that the combination improved median OS and was well tolerated [17].

A retrospective study evaluated the safety and efficacy of the combination of 5-FU, leucovorin, and irinotecan (FOLFIRI) regimens in NEC patients who progressed on the etoposide–platinum combination. Nineteen of the thirty-nine patients who progressed on etoposide–platinum were eligible for FOLFIRI; six patients (31%) had an objective response, and six (31%) had stable disease. The authors concluded that the FOLFIRI regimen is safe and a potentially effective second-line chemotherapy regimen in NEC patients who remain in good condition and with reasonable liver function after the failure of the etoposide–platinum combination [18].

Guided by the data from these studies, we hypothesized that nal-IRI + 5-FU would be a safe and effective second-line option in patients with high-grade NEC who had progressed on a prior platinum-based regimen. We also looked at the role of UGT1A1*28 polymorphism on the efficacy parameters and side effects experienced by patients receiving nanoliposomal encapsulated irinotecan.

## 2. Methods

### 2.1. Patients

Patients with refractory advanced high-grade neuroendocrine cancer of the gastrointestinal tract, of unknown or pancreatic origin (lung primary excluded), and measurable disease per RECIST 1.1 were included. Eligible patients had an Eastern Cooperative Oncology Group (ECOG) performance status of 0–2; adequate renal, hepatic, and bone marrow function; and a life expectancy greater than 12 weeks. Included participants had tissue available for central pathology review and pathologically/histologically confirmed high-grade neuroendocrine tumors defined as Ki-67 proliferative index of 20–100% or evidence of at least 10 mitotic figures per 10 high-powered fields. Patients were excluded if they had known CNS metastases, dihydropyrimidine dehydrogenase (DPD) deficiency, or uncontrolled intercurrent illness. Patients were also excluded if they had received investigational therapy within four weeks of starting study treatment.

Pretreatment of tumor tissue or blood NGS was required before starting therapy. UGT1A1*28 status was also tested. Of the eleven patients enrolled in the trial, one patient received frontline treatment with FOLFOX; one patient received cisplatin and etoposide, two patients received carboplatin, etoposide, and atezolizumab; and seven patients received carboplatin and etoposide. The trial was approved by the Institutional Review Board at Roswell Park Cancer Institute and Ochsner Cancer Center and conducted in accordance with Good Clinical Practice guidelines. All study participants were required to provide written informed consent.

### 2.2. Study Design/Treatment

This open-label, single-arm, multi-center Phase II trial was conducted at Roswell Park Comprehensive Cancer Center and Ochsner Cancer Center between 2019 and 2023. The primary objective of the study was to determine the antitumor efficacy of nanoliposomal irinotecan (nal-IRI) + fluorouracil (5-FU) and leucovorin (LV) in refractory advanced high-grade neuroendocrine cancer of GI, unknown, or pancreatic origin.

The secondary objectives were to determine overall survival (OS), progression-free survival (PFS), safety, and clinical response, which were assessed via changes in tumor burden and quality of life (QoL) resulting from the combination treatment.

Additionally, the exploratory objective was to perform genetic profiling (in pre- and post-treatment samples) to identify biomarkers that may correlate with response. Treatment was administered on an outpatient basis. Nal-IRI and 5-FU/LV were administered intravenously on days 1 and 15 of each 28-day cycle. We planned to enroll 18 patients in stage 1; if we saw five or more responses, we would progress to stage 2 and enroll an additional 19 patients.

### 2.3. Safety Monitoring

Toxicity was evaluated by monitoring adverse events. All patients who received at least one dose of any of the study drugs (nanoliposomal irinotecan, leucovorin, 5-fluorouracil) were evaluated for toxicity.

### 2.4. Efficacy

Computed tomography/magnetic resonance imaging (CT/MRI) scans were performed at screening and every eight weeks after that to assess response. The primary endpoint of the study was objective response rate (ORR) as determined by RECIST 1.1. Secondary endpoints included OS, PFS, quality of life changes, and safety of the combination. The exploratory endpoint was comprehensive molecular profiling for mutations, for which baseline tissue or blood samples were submitted for next-generation sequencing (NGS). Subsequently, on treatment, blood samples were collected for circulating tumor DNA (CtDNA) measurement using Foundation One^®^ Liquid assay (Boston, MA, USA).

### 2.5. Quality of Life (QoL)

We used the EORTC QLQ-C30 (EORTC Quality of Life Group, Brussels, Belgium) to assess QoL [19]. We looked at various parameters to assess health-related quality of life (HRQoL), such as global health status, physical functioning, role functioning, emotional functioning, cognitive functioning, social functioning, financial difficulties, as well as side effects affecting day-to-day activities, including fatigue, nausea/vomiting, pain, dyspnea, insomnia, appetite loss, constipation, and diarrhea.

### 2.6. Statistics

Descriptive statistics (as appropriate: number, percent, mean, median, min, and max) were used to summarize demographic and baseline characteristics. The primary outcome was an objective response rate (ORR) (based on the best overall response within six months of treatment initiation: complete and partial response, defined by the RECIST 1.1 criteria). Objective response was treated as binary data and summarized using frequencies and relative frequencies, with the ORR estimated using an 80% confidence interval obtained using Jeffrey’s prior method.

Overall survival (OS) was defined as the time from the start of treatment until death or last follow-up. Progression-free survival (PFS) was defined as the time from the start of treatment until disease progression, subsequent treatment, death, or last follow-up. OS and PFS were summarized in the overall sample and by evaluable status using standard Kaplan–Meier methods, where estimates of the median were obtained with 95% confidence intervals.

Toxicities and adverse events were summarized by attribution and grade using frequencies and relative frequencies. High-grade (3+) toxicity and adverse event rates were estimated using 90% confidence intervals obtained by Jeffrey’s prior method.

The quality of life (QoL) measures were summarized by timepoint using the mean, median, standard deviation, and IQR. Comparisons between baseline to cycle 1, baseline to cycle 2, and cycle 1 to cycle 2 were made using the sign test. Line plots and mean plots were generated by timepoint. QoL was also assessed by best response (excluding ‘Not Evaluable’ responses) using the mean, median, standard deviation, and IQR. Comparisons were made using the Kruskal–Wallis test. Time-dependent univariate Cox regression modeling was performed to measure associations between survival outcomes (OS/PFS) and each quality-of-life measure. Hazard ratios, 95% confidence intervals, and *p*-values were reported.

All analyses were conducted in SAS v9.4 (Cary, NC) at a significance level of 0.05.

Sample size calculation: The study had Simon’s minimax design, with a plan to enroll 18 patients in stage 1. In stage 1, *n*1 = 18 was to be enrolled, and the response was evaluated. If T1 = 3 or fewer responses were observed, the study would terminate, and the treatment would not be considered promising; otherwise, an additional *n*2 = 19 patients would be enrolled in stage 2. If T = 8 or fewer of the total *n* = *n*1 + *n*2 = 37 patients had a response, the treatment would not be considered promising; otherwise, the treatment would be considered promising for further study. Unfortunately, due to a lack of funding, the study was closed to accrual after enrolling 11 patients.

## 3. Results

### 3.1. Patient Characteristics

**Figure 1:**
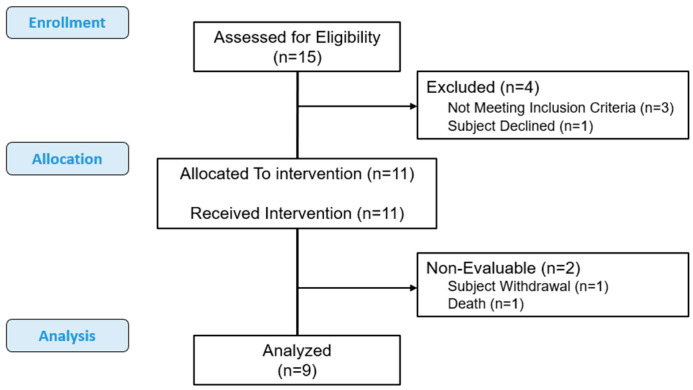
CONSORT diagram.

A total of eleven patients were enrolled at two sites in this study between 2019 and 2023, of whom nine were considered evaluable (Figure 1). The study closed early due to a lack of funding support. Table 1 summarizes the demographic and clinical characteristics of all enrolled patients. Seven (63.6%) of the patients were male; the median age was 66.7 years (range: 50.0–87.8 years), and the median Ki-67 was 90% (range: 50–100%), with the primary site of NEC as follows: three colorectal, two esophageal, two ampullary, and the remaining four at other sites. Eight of the enrolled patients had liver metastases. Five patients were heterozygous for UGT1A1*28 polymorphism.

### 3.2. Treatment Response

**Figure 2:**
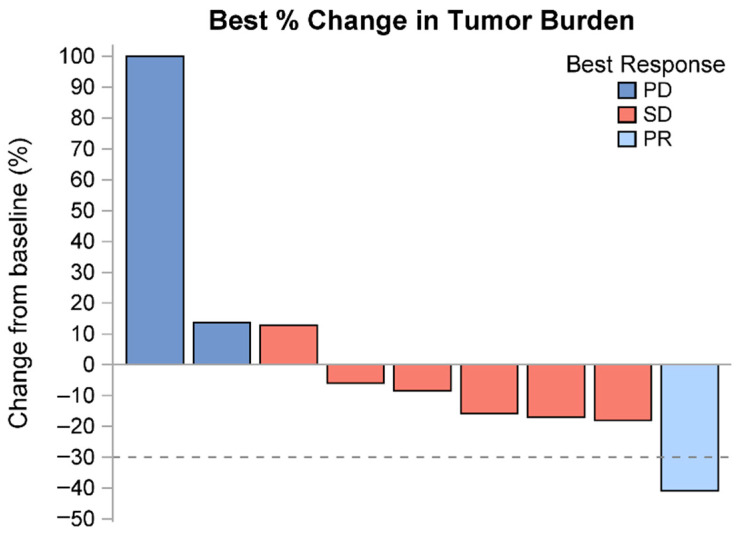
Waterfall plot.

Of the nine evaluable patients, the best response seen in our study was a partial response in one patient. Stable disease was seen in six patients, while two patients had progressive disease. Eight patients were eventually taken off treatment for progression, which led to death, whereas one patient was sent to a hospice. During treatment, five patients required dose reductions and interruptions due to treatment toxicity. After going off study treatment, one patient had a small bowel resection, two patients ended up needing radiation therapy, and five patients received subsequent chemotherapy (Table 2 and Figure 2).

### 3.3. Treatment Efficacy-Survival

**Figure 3:**
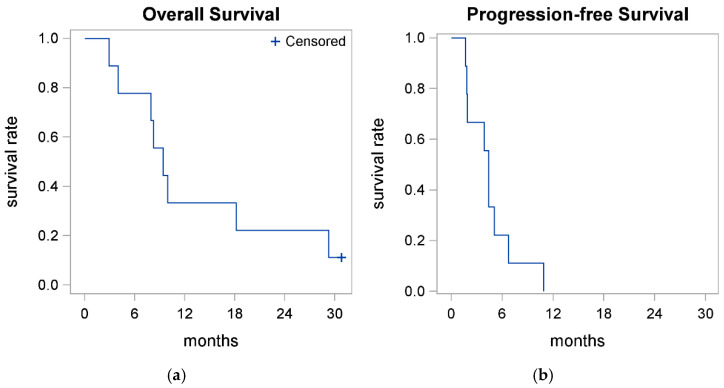
Kaplan–Meier curves for (**a**) OS and (**b**) PFS (evaluable patients).

We evaluated the overall (OS) and progression-free survival (PFS) of all evaluable patients on nal-IRI and 5-FU. The median OS was 9.4 months (95% CI 2.9–29.3 mo) after a median follow-up of 30.8 months, and the median PFS was 4.4 months (95% CI 1.7–6.7 mo). The 1-year OS rate was 0.33 (95% CI 0.08–0.62). There were a total of 10 deaths during the study period. The one patient still alive has been on follow-up for 30.8 months at the time of the data cutoff (Table 3 and Figure 3).

### 3.4. Exploratory Mutational Analysis

The most common somatic mutations were TP53 (88.9%), CHEK2 (88.9%), APC (33.3%), and NF1 (11.1%), and they mainly remained detectable throughout treatment. Subgroup analysis (by mutational status) demonstrated that patients harboring CHEK2 mutation had longer PFS (*p* = 0.005). APC mutation status was also associated with longer PFS (*p* = 0.013). Cox regression models also examine the association between expression and survival. A moderate association between P53 expression and OS (HR = 1.02; 95% CI 0.99–1.04; *p* = 0.065) was seen.

The supplementary section reports OS and PFS by mutation status of four genes: APC, CHEK2, NF1, and TP53 (Table S1 and Figure S1).

Treatment efficacy/survival based on UGT1A1*28 polymorphism status:

Out of eleven patients, ten patients had UGT1A1 status reported. Five were UGT1A1 normal variants, and the other five had heterozygous UGT1A1*28 polymorphism. The median OS for the normal variant was 8.0 mo (95% CI 2.3–18.2) and was 9.4 mo (0.4 not reached) for UGT1A1*28 heterozygous patients, although this was not statistically significant (*p* = 0.41). The median PFS for the normal variant was 1.9 mo (95% CI 0.5–10.9), whereas it was 3.9 mo (95% CI 0.4–4.4) for UGT1A1 heterozygous patients (*p* = 0.51) (Table S2 and Figure S2).

### 3.5. Safety

The combination of nal-IRI/5-FU was well tolerated. Among all treated patients (*n* = 11), nine (82%) had a treatment-related adverse event (AE), with seven (64%) having grade 3 or higher AEs. The most common AEs were diarrhea (45%), nausea (45%), vomiting (45%), and fatigue (45%). No treatment discontinuation occurred due to side effects. The most common grade 3+ toxicities were diarrhea (18.2%), sepsis (18.2%), and neutropenia (18.2%) (Table 4 and Table S3).

Based on the UGT1A1 status, five patients with normal UGT1A1 and four with heterozygous UGT1A1*28 status had side effects, of which four normal variants and four heterozygous variants each had grade 3+ adverse events. Four normal variants had SAE, while only two heterozygous variants had SAE. The differences in the side-effect profile were not significant between heterozygous variants and normal variants (Table S4).

### 3.6. Quality of Life

The EORTC QLG Core Questionnaire (EORTC QLQ-C30) was used to assess QoL [20]. Quality of life parameters, including global health status and physical/role/emotional/cognitive and social functioning were maintained across different timepoints: baseline, cycle 1 Day 1, and cycle 2 Day 1. The decline in role functioning (HR 0.946; 95% CI 0.898–0.997; *p* = 0.0369) and increased fatigue (HR 1.034; 95% CI 1.001–1.068; *p* = 0.046) and dyspnea (HR 1.034; 95%CI 1.000–1.069; *p* = 0.048) showed a significant association with poor OS. Pain (HR 1.031; 95% CI 0.997–1.067; *p* = 0.076) and decline in physical (HR 0.957; 95% CI 0.915–1.000; *p* = 0.05) and social functioning (HR 0.97; 95% CI 0.933–1.001; *p* = 0.06) showed a moderate association with OS.

A decline in global health status (HR 0.958; 95% CI 0.920–0.997; *p* = 0.03), role functioning (HR 0.910; 95% CI 0.837–0.990, *p* = 0.028), emotional functioning (HR 0.945; 95% CI 0.897–0.995; *p* = 0.032), and social functioning (HR 0.948; 95% CI 0.905–0.994; *p* = 0.026) and increased pain (HR 1.084; 95% CI 1.014–1.159; *p* = 0.018) showed a statistically significant association with PFS. A decline in physical functioning (HR 0.929; 95% CI 0.863–1.001; *p* = 0.052) and the presence of fatigue (HR 1.030; 95% CI 1.000–1.060; *p* = 0.051) and dyspnea (HR 1.104; 95% CI 0.992–1.229; *p* = 0.07) showed a moderate association with PFS. Complete QoL data measures by visit, best response, and association with survival measures are provided in Tables S5–S7 in the Supplementary Materials.

## 4. Discussion

Our study was a single-arm, open-label, multi-center, phase II study evaluating the efficacy of nano-liposomal irinotecan combination with 5-fluorouracil and leucovorin in locally advanced and unresectable or metastatic refractory extrapulmonary neuroendocrine carcinoma. We found that a combination of nal-IRI and 5-FU was an acceptable second-line option for treatment with manageable toxicity. We also found no differences in the efficacy or toxicity profile of patients based on their UGT1A1*28 status.

Although a promising field, research on high-grade neuroendocrine carcinomas remains scarce; thus, there are limited therapeutic options, especially in the second-line setting. The recent NET-02 trial compared nal-IRI/5-FU with docetaxel as second-line agents for poorly differentiated extrapulmonary NECs and showed that only nal-IRI/5-FU reached the primary endpoint of the six-month PFS rate and exceeded the threshold for efficacy. The quality of life was only maintained in the combination arm [20]. However, in the final analysis, no difference was observed in the objective response rate, median OS, or PFS between the nal-IRI/5FU arm and the docetaxel arm [21]. The objective response rate was 11.1% in the combination arm, which was exactly similar to the ORR in our study. Additionally, our 6-month PFS rate was 22% compared to the 29.6% (lower 95% CI 15.68) observed in the NET-02 trial’s final analysis.

Irinotecan is a pro-drug that metabolizes into an active metabolite SN-38, a potent inhibitor of Topoisomerase 1 (TOP1). This inhibition leads to the interruption of cell division and subsequent cell death, perpetuating its antitumor properties [22,23]. Liposomal encapsulation improves the half-life of irinotecan and increases the time in circulation before conversion to its active metabolite SN-38, allowing gradual release and more targeted deposition in tumor tissues [14]. Liposomal encapsulation also decreases the dose needed to achieve therapeutic SN-38 levels in tumor xenograft models by up to five times [24]. This reduced dosage requirement leads to a better toxicity profile without adversely affecting efficacy outcomes [25].

The UGT1A1*28 allele has been associated with irinotecan toxicity due to poor metabolism [12]. However, liposomal encapsulation appears to protect against toxicity. In a recent study, UGT1A1*28 status was not found to be a significant predictor of SN-38 levels following a nal-IRI dose. It appears that nal-IRI had a slower irinotecan release rate, avoiding rapid spikes of plasma SN-38, which is attributed to the toxicity seen in this population [15]. However, despite no differences in side effects observed in the above study and our results, dose adjustments are still recommended for patients homozygous for UGT1A1*28 [13].

We performed mutational analysis and identified the TP53, CHEK2, APC, and NF1 genes in decreasing order of prevalence in our sample set. The presence of CHEK2 and APC mutations was associated with longer PFS. CHEK2, a tumor suppressor gene that plays a crucial role in DNA damage response, has been identified in some case reports in neuroendocrine carcinoma and small cell carcinoma but has not been studied in association with outcomes in this population [26,27]. However, one study has demonstrated that irinotecan-containing therapy improves PFS more than other treatment regimens in patients with pancreatic ductal adenocarcinoma harboring CHEK2/ATM mutations (germline/somatic) [28]. This information, in conjunction with our results, suggests that CHEK2 mutational status can aid in the selection of treatment regimens containing irinotecan [29].

Quality-of-life was assessed in our trial and measured by the EORTC-QLQ C30 tool. Improved OS was associated with better role and physical functioning, less fatigue, and dyspnea. Similarly, better global health status, physical functioning, role functioning, emotional functioning, and social functioning, along with less fatigue and pain, were associated with improved PFS. Given that the combination regimen maintained the quality of life of refractory NEC patients while showing preliminary signs of efficacy, it warrants exploration in the first-line setting.

The strength of our study lies in that it is the first study of its kind to provide a multifaceted review of different aspects of nal-IRI treatment in NEC patients—efficacy, safety, and their correlation with the UGT1A1*28 allele variation. We also looked at the comprehensive genomic profile of these patients and correlated treatment outcomes with somatic mutations. We shed light on not only its efficacy through OS and PFS but also its effect on quality of life and the toxicity profile.

One of the limitations of our study is the small sample size due to the early closure of the trial due to a lack of funding. Another limitation is the lack of a control arm given the unique patient population and sample size. Furthermore, we did not have any homozygous UGT1A1*28 patients in our study. Our study cannot conclusively prove if UGT1A1*28 variations, the liposomal encapsulation of irinotecan, or the lack of sufficient patients led to the lack of difference in adverse events or survival observed in patients with UGT1A1 polymorphisms.

## 5. Conclusions and Future Directions

The results of our study are in concordance with previous studies exploring nal-IRI in NECs and demonstrate that the combination of nanoliposomal irinotecan and 5-fluorouracil is safe and effective in refractory extrapulmonary NECs. Evidence on the efficacy of second-line treatment of NECs remains limited, and our study aims to add to this knowledge gap. Larger studies should explore this combination as a backbone to investigate new therapeutic avenues for managing high-grade NECs, both in the refractory and frontline settings.

## Figures and Tables

**Table 1:** Demographic and clinical characteristics of all patients.

		Non-Evaluable	Evaluable	Overall
Overall	N	2 (18.2)	9 (81.8)	11 (100%)
Age	Mean/Std/N	71.5/0.5/2	64.8/11.6/9	66.0/10.7/11
	Median/Min/Max	71.5/71.2/71.8	66.1/50.0/87.8	66.7/50.0/87.8
Sex	Male	1 (50.0%)	6 (66.7%)	7 (63.6%)
	Female	1 (50.0%)	3 (33.3%)	4 (36.4%)
Race	White	1 (50.0%)	8 (88.9%)	9 (81.8%)
	Asian	1 (50.0%)		1 (9.1%)
	Not Reported		1 (11.1%)	1 (9.1%)
Ethnicity	Non-Hispanic	2 (100.0%)	7 (77.8%)	9 (81.8%)
	Not Reported		2 (22.2%)	2 (18.2%)
BMI	Mean/Std/N	22.5/2.4/2	28.2/5.9/9	27.1/5.8/11
	Median/Min/Max	22.5/20.7/24.2	26.1/22.1/37.6	25.0/20.7/37.6
Diabetes	No	2 (100.0%)	9 (100.0%)	11 (100.0%)
Dyspnea	No	2 (100.0%)	9 (100.0%)	11 (100.0%)
HTN	No	2 (100.0%)	6 (66.7%)	8 (72.7%)
	Yes		3 (33.3%)	3 (27.3%)
Cancer History	No	1 (50.0%)	8 (88.9%)	9 (81.8%)
	Yes	1 (50.0%)	1 (11.1%)	2 (18.2%)
Weight Loss	No	2 (100.0%)	9 (100.0%)	11 (100.0%)
ECOG	0		5 (55.6%)	5 (45.5%)
	1	2 (100.0%)	4 (44.4%)	6 (54.5%)
Disease Site	Esophagus		2 (22.2%)	2 (18.2%)
	Ampulla		2 (22.2%)	2 (18.2%)
	Pancreas		1 (11.1%)	1 (9.1%)
	GI Tract		1 (11.1%)	1 (9.1%)
	Colon		1 (11.1%)	1 (9.1%)
	Rectum		1 (11.1%)	1 (9.1%)
	Cecum		1 (11.1%)	1 (9.1%)
	Cervix	1 (50.0%)		1 (9.1%)
	Not Reported	1 (50.0%)		1 (9.1%)
Stage	IV	2 (100.0%)	9 (100.0%)	11 (100.0%)
Grade	Grade 3	2 (100.0%)	8 (88.9%)	10 (90.9%)
	Grade X		1 (11.1%)	1 (9.1%)
Tumor Size (mm)	Mean/Std/N	146.5/136.5/2	68.6/31.8/8	84.2/62.7/10
	Median/Min/Max	146.5/50.0/243.0	77.0/10.0/110.0	77.0/10.0/243.0
Ki67 (%)	Mean/Std/N	95.0/7.1/2	82.5/16.7/8	85.0/15.8/10
	Median/Min/Max	95.0/90.0/100.0	90.0/50.0/95.0	90.0/50.0/100.0
UGT1A1-28 Allele	Negative	1 (50.0%)	4 (44.4%)	5 (45.5%)
	Heterozygous	1 (50.0%)	4 (44.4%)	5 (45.5%)
	Not Reported		1 (11.1%)	1 (9.1%)
Mets: Bone	No	2 (100.0%)	8 (88.9%)	10 (90.9%)
	Yes		1 (11.1%)	1 (9.1%)
Mets: Brain	No	2 (100.0%)	9 (100.0%)	11 (100.0%)
Mets: Liver	No	1 (50.0%)	2 (22.2%)	3 (27.3%)
	Yes	1 (50.0%)	7 (77.8%)	8 (72.7%)
Mets: LN	No	1 (50.0%)	8 (88.9%)	9 (81.8%)
	Yes	1 (50.0%)	1 (11.1%)	2 (18.2%)
Mets: Lung	No	2 (100.0%)	9 (100.0%)	11 (100.0%)
# Prior Chemo Regimens	1	2 (100.0%)	5 (62.5%)	7 (70.0%)
	2		3 (37.5%)	3 (30.0%)
Prior Chemo	Yes	2 (100.0%)	9 (100.0%)	11 (100.0%)
Prior Radiation	No	2 (100.0%)	8 (88.9%)	10 (90.9%)
	Yes		1 (11.1%)	1 (9.1%)
Prior Surgery	No	2 (100.0%)	9 (100.0%)	11 (100.0%)
TP53 Mutation	No		1 (11.1%)	1 (9.1%)
	Yes	2 (100.0%)	8 (88.9%)	10 (90.9%)
CHEK2 Mutation	No	2 (100.0%)	1 (11.1%)	3 (27.3%)
	Yes		8 (88.9%)	8 (72.7%)
APC Mutation	No	2 (100.0%)	6 (66.7%)	8 (72.7%)
	Yes		3 (33.3%)	3 (27.3%)
NF1 Mutation	No	1 (50.0%)	8 (88.9%)	9 (81.8%)
	Yes	1 (50.0%)	1 (11.1%)	2 (18.2%)
TP53 Expression	Mean/Std/N	86.0/9.7/2	18.6/34.0/9	30.9/40.9/11
	Median/Min/Max	86.0/79.2/92.9	0.7/0.0/88.9	1.6/0.0/92.9
CHEK2 Expression	Mean/Std/N	0.0/0.0/2	6.1/16.4/9	5.0/14.8/11
	Median/Min/Max	0.0/0.0/0.0	0.4/0.0/49.7	0.3/0.0/49.7
NF1 Expression	Mean/Std/N	24.2/34.3/2	0.0/0.0/9	4.4/14.6/11
	Median/Min/Max	24.2/0.0/48.5	0.0/0.0/0.0	0.0/0.0/48.5

**Table 2:** Summary of response and treatment data.

		Non-Evaluable	Evaluable	Overall
Overall	N	2 (18.2)	9 (81.8)	11 (100%)
Best Response	PR		1 (11.1%)	1 (9.1%)
	SD		6 (66.7%)	6 (54.5%)
	PD	1 (50.0%)	2 (22.2%)	3 (27.3%)
	Not Evaluable	1 (50.0%)		1 (9.1%)
Maximum Change in Tumor Burden (%)	Mean/Std/N		2.2/40.2/9	2.2/40.2/9
	Median/Min/Max		−8.4/−40.8/100.0	−8.4/−40.8/100.0
Reason Off Treatment	Progression	1 (50.0%)	8 (88.9%)	9 (81.8%)
	Death	1 (50.0%)		1 (9.1%)
	Hospice		1 (11.1%)	1 (9.1%)
Reason Off Study	On Study		1 (11.1%)	1 (9.1%)
	Death	1 (50.0%)	8 (88.9%)	9 (81.8%)
	Hospice	1 (50.0%)		1 (9.1%)
Dose Reductions	No	2 (100.0%)	4 (37.5%)	6 (54.5%)
	Yes		5 (62.5%)	5 (45.5%)
Dose Interruptions	No	1 (50.0%)	4 (37.5%)	5 (45.5%)
	Yes	1 (50.0%)	5 (62.5%)	6 (54.5%)
Subsequent Chemotherapy	No	2 (100.0%)	4 (37.5%)	6 (54.5%)
	Yes		5 (62.5%)	5 (45.5%)
Subsequent RT	No	2 (100.0%)	7 (77.8%)	9 (81.8%)
	Yes		2 (22.2%)	2 (18.2%)

**Table 3:** OS and PFS in evaluable patients.

	6-Month Rate	12-Month Rate	Median	Median Follow-Up
OS (Evaluable)	0.78 (0.36, 0.94)	0.33 (0.08, 0.62)	9.4 (2.9, 29.3)	30.8
PFS (Evaluable)	0.22 (0.03, 0.51)	0.00 (0.01, 0.39)	4.4 (1.7, 6.7)	-

**Table 4:** All adverse events that occurred with >10% frequency in all patients.

	Grade 3+	Any AE
Diarrhea	2 (18.2%)	5 (45.5%)
Nausea	0 (0.0%)	5 (45.5%)
Vomiting	0 (0.0%)	5 (45.5%)
Fatigue	1 (9.1%)	5 (45.5%)
Neutropenia	2 (18.2%)	3 (27.3%)
Dysgeusia	0 (0.0%)	2 (18.2%)
Sepsis	2 (18.2%)	2 (18.2%)

## Data Availability

Data used in the study are not publicly available due to containing information that could compromise the privacy of research participants but are available from Supplementary Materials.

## Supplementary Materials

The following supporting information can be downloaded at https://www.mdpi.com/article/10.3390/cancers17020224/s1. Figure S1: Kaplan–Meier survival curves by mutational status: (a) APC and (b) CHEK2; Figure S2: Kaplan–Meier survival curves by UGT1A1 status. Positive: heterozygous UGT1A1*28 polymorphism, Negative: normal UGT1A1; Table S1: OS and PFS by mutation status; Table S2: OS and PFS by UGT1A1 polymorphism status: Positive: heterozygous UGT1A1*28 polymorphism, Negative: normal UGT1A1; Table S3: Summary of Baseline QoL Measures by Best Response; Table S4: All adverse events by UGT1A1 polymorphism status: Positive: heterozygous UGT1A1*28 polymorphism, Negative: normal UGT1A1; Table S5: Summary of Quality of Life (QoL) Measures by Visit; Table S6: Summary of Baseline QoL Measures by Best Response; Table S7: Association between QoL Measures and Survival (OS/PFS): Univariate Cox Regression Models (Time-Dependent).
