# Supplementary material for: Phase II Study of Nanoliposomal Irinotecan (Nal-IRI) with 5-Fluorouracil and Leucovorin in Refractory Advanced High-Grade Neuroendocrine Cancer of Gastroenteropancreatic (GEP) or Unknown Origin
Source: Cancers (Basel). 2025 Jan 12;17(2):224. doi: 10.3390/cancers17020224 (PMC11763628; doi:10.3390/cancers17020224)
Supplement: Supplementary file 1 [file cancers-17-00224-s001.zip › cancers-3386287-supplementary.pdf]

Supplementary tables and figures:

Figure S1: Kaplan-Meier survival curves by mutational status: (a) APC and (b) CHEK2

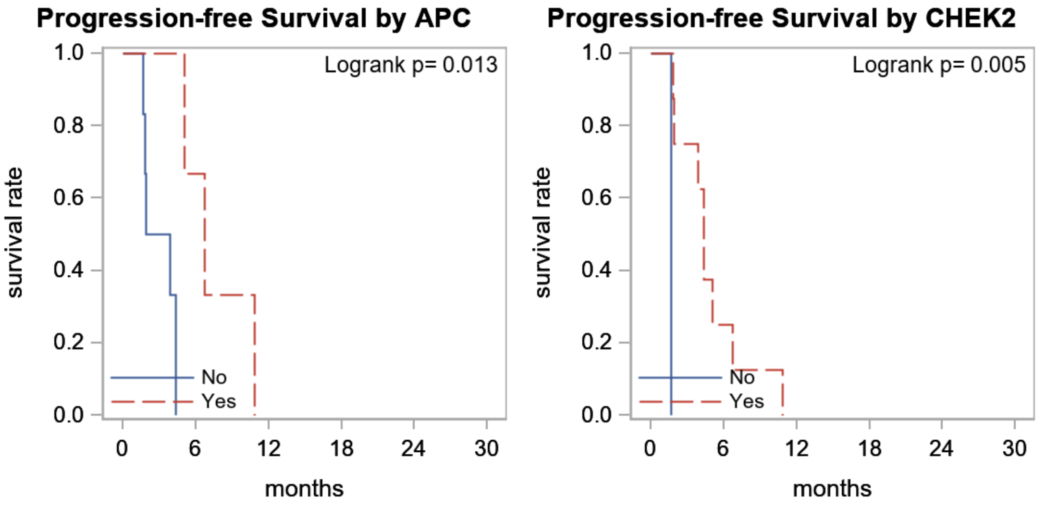

Figure S2: Kaplan-Meier survival curves by UGT1A1 status. Positive: heterozygous UGT1A1*28 polymorphism, Negative: normal UGT1A1

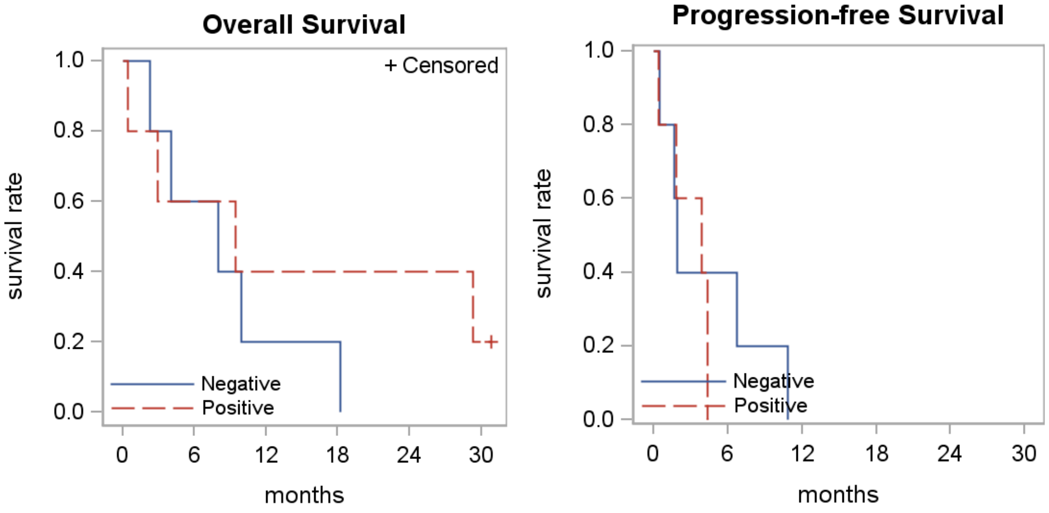

Table S1: OS and PFS by mutation status

			6-month Rate (95% CI)	12-month Rate (95% CI)	Median (95% CI)
OS	APC	No	0.67 (0.19, 0.90)	0.33 (0.05, 0.68)	9.7 (2.9, NR)
		Yes	1.00 (1.00, 1.00)	0.33 (0.01, 0.77)	8.2 (8.0, 18.2)
	CHEK2	No	1.00 (1.00, 1.00)	0.00 (1.00, 1.00)	10.0 (NR, NR)
		Yes	0.75 (0.31, 0.93)	0.38 (0.09, 0.67)	8.8 (2.9, 29.3)
	NF1	No	0.75 (0.31, 0.93)	0.38 (0.09, 0.67)	9.1 (2.9, 29.3)
		Yes	1.00 (1.00, 1.00)	0.00 (1.00, 1.00)	9.4 (NR, NR)
	TP53	No	1.00 (1.00, 1.00)	0.00 (1.00, 1.00)	8.0 (NR, NR)
		Yes	0.75 (0.31, 0.93)	0.38 (0.09, 0.67)	9.7 (2.9, 29.3)
PFS	APC	No	0.00 (0.05, 0.68)		2.9 (1.7, 4.4)
		Yes	0.67 (0.05, 0.95)	0.00 (0.01, 0.77)	6.7 (5.1, 10.9)
	CHEK2	No	0.00 (1.00, 1.00)		1.7 (NR, NR)
		Yes	0.25 (0.04, 0.56)	0.00 (0.01, 0.42)	4.4 (1.8, 6.7)
	NF1	No	0.25 (0.04, 0.56)	0.00 (0.01, 0.42)	4.1 (1.7, 6.7)
		Yes	0.00 (1.00, 1.00)		4.4 (NR, NR)
	TP53	No	1.00 (1.00, 1.00)	0.00 (1.00, 1.00)	6.7 (NR, NR)

Table S2: OS and PFS by UGT1A1 polymorphism status: Positive: heterozygous UGT1A1*28 polymorphism, Negative: normal UGT1A1

		6-month Rate (95% CI)	12-month Rate (95% CI)	Median (95% CI)	Median Follow-up (Range)	Sample
OS	Total	0.60 (0.25, 0.83)	0.30 (0.07, 0.58)	8.7 (0.4, 18.2)	30.8 (30.8, .)	E=9 C=1 T=10
	Negative	0.60 (0.13, 0.88)	0.20 (0.01, 0.58)	8.0 (2.3, 18.2)	. (., .)	E=5 C=0 T=5
	Positive	0.60 (0.13, 0.88)	0.40 (0.05, 0.75)	9.4 (0.4, NR)	30.8 (30.8, .)	E=4 C=1 T=5
PFS	Total	0.20 (0.03, 0.47)	0.00 (0.00, 0.36)	2.9 (0.4, 4.4)		E=10 C=0 T=10
	Negative	0.40 (0.05, 0.75)	0.00 (0.00, 0.58)	1.9 (0.5, 10.9)		E=5 C=0 T=5
	Positive	0.00 (0.00, 0.75)	0.00 (0.00, 0.75)	3.9 (0.4, 4.4)		E=5 C=0 T=5

Table S3: Summary of Baseline QoL Measures by Best Response

		PR	SD	PD	P-Value
Overall	N	1 (10.0)	5 (50.0)	4 (40.0)	
Global Health Status	Mean/Std/N	75.00/./1	90.00/9.13/5	79.17/31.55/4	0.416
	Median (IQR)	75.00/0.00	83.33/16.67	91.67/41.67	
Physical Functioning	Mean/Std/N	100.00/./1	90.67/5.96/5	81.67/32.38/4	0.442
	Median (IQR)	100.00/0.00	86.67/6.67	96.67/36.67	
Role Functioning	Mean/Std/N	111.11/./1	106.67/9.94/5	102.78/16.67/4	0.836
	Median (IQR)	111.11/0.00	111.11/0.00	111.11/16.67	
Emotional Functioning	Mean/Std/N	100.00/./1	88.33/13.94/5	77.08/24.88/4	0.421
	Median (IQR)	100.00/0.00	91.67/16.67	83.33/29.17	
Cognitive Functioning	Mean/Std/N	83.33/./1	86.67/13.94/5	87.50/25.00/4	0.729
	Median (IQR)	83.33/0.00	83.33/16.67	100.00/25.00	
Social Functioning	Mean/Std/N	83.33/./1	93.33/9.13/5	91.67/16.67/4	0.606
	Median (IQR)	83.33/0.00	100.00/16.67	100.00/16.67	
Fatigue	Mean/Std/N	0.00/./1	15.56/14.91/5	33.33/47.14/4	0.612
	Median (IQR)	0.00/0.00	22.22/22.22	16.67/66.67	
Nausea/Vomiting	Mean/Std/N	0.00/./1	13.33/7.45/5	12.50/25.00/4	0.379
	Median (IQR)	0.00/0.00	16.67/0.00	0.00/25.00	
Pain	Mean/Std/N	16.67/./1	20.00/13.94/5	16.67/33.33/4	0.600
	Median (IQR)	16.67/0.00	16.67/16.67	0.00/33.33	
Dyspnea	Mean/Std/N	0.00/./1	6.67/14.91/5	8.33/16.67/4	0.869
	Median (IQR)	0.00/0.00	0.00/0.00	0.00/16.67	
Insomnia	Mean/Std/N	0.00/./1	13.33/18.26/5	33.33/47.14/4	0.631
	Median (IQR)	0.00/0.00	0.00/33.33	16.67/66.67	
Appetite Loss	Mean/Std/N	0.00/./1	6.67/14.91/5	41.67/50.00/4	0.405
	Median (IQR)	0.00/0.00	0.00/0.00	33.33/83.33	

		PR	SD	PD	P-Value
Constipation	Mean/Std/N	0.00/./1	6.67/14.91/5	8.33/16.67/4	0.869
	Median (IQR)	0.00/0.00	0.00/0.00	0.00/16.67	
Diarrhea	Mean/Std/N	0.00/./1	6.67/14.91/5	8.33/16.67/4	0.869
	Median (IQR)	0.00/0.00	0.00/0.00	0.00/16.67	
Financial Difficulties	Mean/Std/N	0.00/./1	13.33/18.26/5	8.33/16.67/4	0.725
	Median (IQR)	0.00/0.00	0.00/33.33	0.00/16.67	

Table S4: All adverse events by UGT1A1 polymorphism status: Positive: heterozygous UGT1A1*28 polymorphism, Negative: normal UGT1A1

		Negative	Positive	P-value
Overall	N	5 (50.0)	5 (50.0)	
Any AE	No		1 (20.0%)	0.292
	Yes	5 (100.0%)	4 (80.0%)	
Any Grade 3+ AE	No	1 (20.0%)	1 (20.0%)	1.000
	Yes	4 (80.0%)	4 (80.0%)	
Any Treatment Related AE	No		1 (20.0%)	0.292
	Yes	5 (100.0%)	4 (80.0%)	
Any Grade 3+ TRAE	No	2 (40.0%)	1 (20.0%)	0.490
	Yes	3 (60.0%)	4 (80.0%)	
SAE	No	1 (20.0%)	3 (60.0%)	0.197
	Yes	4 (80.0%)	2 (40.0%)	

Table S5: Summary of Quality of Life (QoL) Measures by Visit

		Baseline	Cycle 1, Day 1	Cycle 2, Day 1	Sign Test P-value *(Baseline – Cycle 1)*	Sign Test P-value *(Baseline – Cycle 2)*	Sign Test P-value *(Cycle 1 – Cycle 2)*
Overall	N	11 (36.7)	10 (33.3)	9 (30.0)			
Global Health Status	Mean/Std/N	81.82/20.69/11	79.17/23.65/10	83.33/17.18/9	1.0000	0.1250	0.2500
	Median (IQR)	83.33/25.00	83.33/25.00	83.33/25.00			
Physical Functioning	Mean/Std/N	85.45/20.83/11	80.67/31.65/10	95.56/5.77/9	1.0000	1.0000	1.0000
	Median (IQR)	93.33/13.33	93.33/13.33	100.00/6.67			
Role Functioning	Mean/Std/N	106.06/11.51/11	101.11/21.24/10	104.94/9.80/9	1.0000	0.5000	1.0000
	Median (IQR)	111.11/0.00	111.11/11.11	111.11/11.11			
Emotional Functioning	Mean/Std/N	80.30/23.65/11	79.17/26.43/10	94.44/8.33/9	1.0000	0.0625	0.2500
	Median (IQR)	83.33/33.33	87.50/25.00	100.00/8.33			
Cognitive Functioning	Mean/Std/N	87.88/16.82/11	83.33/15.71/10	94.44/8.33/9	1.0000	0.6250	0.6875
	Median (IQR)	100.00/16.67	83.33/33.33	100.00/16.67			
Social Functioning	Mean/Std/N	87.88/16.82/11	81.67/30.88/10	90.74/12.11/9	1.0000	0.6250	0.3750
	Median (IQR)	100.00/16.67	100.00/16.67	100.00/16.67			
Fatigue	Mean/Std/N	22.22/29.81/11	24.44/33.46/10	19.75/13.35/9	1.0000	1.0000	1.0000
	Median (IQR)	22.22/33.33	16.67/22.22	22.22/22.22			
Nausea/Vomiting	Mean/Std/N	10.61/15.41/11	5.00/8.05/10	18.52/19.44/9	1.0000	1.0000	1.0000
	Median (IQR)	0.00/16.67	0.00/16.67	16.67/16.67			
Pain	Mean/Std/N	19.70/20.84/11	28.33/34.29/10	7.41/12.11/9	1.0000	0.5000	0.5000
	Median (IQR)	16.67/33.33	16.67/50.00	0.00/16.67			
Dyspnea	Mean/Std/N	9.09/15.57/11	16.67/32.39/10	3.70/11.11/9	1.0000	.	.
	Median (IQR)	0.00/33.33	0.00/33.33	0.00/0.00			
Insomnia	Mean/Std/N	21.21/30.81/11	23.33/35.31/10	7.41/14.70/9	1.0000	1.0000	.
	Median (IQR)	0.00/33.33	0.00/33.33	0.00/0.00			
Appetite Loss	Mean/Std/N	18.18/34.52/11	13.33/32.20/10	11.11/16.67/9	.	1.0000	.
	Median (IQR)	0.00/33.33	0.00/0.00	0.00/33.33			
Constipation	Mean/Std/N	6.06/13.48/11	10.00/16.10/10	11.11/16.67/9	.	.	.
	Median (IQR)	0.00/0.00	0.00/33.33	0.00/33.33			
Diarrhea	Mean/Std/N	9.09/15.57/11	6.67/14.05/10	18.52/24.22/9	1.0000	0.5000	1.0000

		Baseline	Cycle 1, Day 1	Cycle 2, Day 1	Sign Test P-value *(Baseline – Cycle 1)*	Sign Test P-value *(Baseline – Cycle 2)*	Sign Test P-value *(Cycle 1 – Cycle 2)*
	Median (IQR)	0.00/33.33	0.00/0.00	0.00/33.33			
Financial Difficulties	Mean/Std/N	12.12/16.82/11	13.33/28.11/10	3.70/11.11/9	1.0000	0.5000	1.0000
	Median (IQR)	0.00/33.33	0.00/0.00	0.00/0.00			

Table S6: Summary of Baseline QoL Measures by Best Response

		PR	SD	PD	P-Value
Overall	N	1 (10.0)	5 (50.0)	4 (40.0)	
Global Health Status	Mean/Std/N	75.00/./1	90.00/9.13/5	79.17/31.55/4	0.416
	Median (IQR)	75.00/0.00	83.33/16.67	91.67/41.67	
Physical Functioning	Mean/Std/N	100.00/./1	90.67/5.96/5	81.67/32.38/4	0.442
	Median (IQR)	100.00/0.00	86.67/6.67	96.67/36.67	
Role Functioning	Mean/Std/N	111.11/./1	106.67/9.94/5	102.78/16.67/4	0.836
	Median (IQR)	111.11/0.00	111.11/0.00	111.11/16.67	
Emotional Functioning	Mean/Std/N	100.00/./1	88.33/13.94/5	77.08/24.88/4	0.421
	Median (IQR)	100.00/0.00	91.67/16.67	83.33/29.17	
Cognitive Functioning	Mean/Std/N	83.33/./1	86.67/13.94/5	87.50/25.00/4	0.729
	Median (IQR)	83.33/0.00	83.33/16.67	100.00/25.00	
Social Functioning	Mean/Std/N	83.33/./1	93.33/9.13/5	91.67/16.67/4	0.606
	Median (IQR)	83.33/0.00	100.00/16.67	100.00/16.67	
Fatigue	Mean/Std/N	0.00/./1	15.56/14.91/5	33.33/47.14/4	0.612
	Median (IQR)	0.00/0.00	22.22/22.22	16.67/66.67	
Nausea/Vomiting	Mean/Std/N	0.00/./1	13.33/7.45/5	12.50/25.00/4	0.379
	Median (IQR)	0.00/0.00	16.67/0.00	0.00/25.00	
Pain	Mean/Std/N	16.67/./1	20.00/13.94/5	16.67/33.33/4	0.600
	Median (IQR)	16.67/0.00	16.67/16.67	0.00/33.33	
Dyspnea	Mean/Std/N	0.00/./1	6.67/14.91/5	8.33/16.67/4	0.869
	Median (IQR)	0.00/0.00	0.00/0.00	0.00/16.67	
Insomnia	Mean/Std/N	0.00/./1	13.33/18.26/5	33.33/47.14/4	0.631
	Median (IQR)	0.00/0.00	0.00/33.33	16.67/66.67	
Appetite Loss	Mean/Std/N	0.00/./1	6.67/14.91/5	41.67/50.00/4	0.405
	Median (IQR)	0.00/0.00	0.00/0.00	33.33/83.33	
Constipation	Mean/Std/N	0.00/./1	6.67/14.91/5	8.33/16.67/4	0.869
	Median (IQR)	0.00/0.00	0.00/0.00	0.00/16.67	
Diarrhea	Mean/Std/N	0.00/./1	6.67/14.91/5	8.33/16.67/4	0.869
	Median (IQR)	0.00/0.00	0.00/0.00	0.00/16.67	
Financial Difficulties	Mean/Std/N	0.00/./1	13.33/18.26/5	8.33/16.67/4	0.725
	Median (IQR)	0.00/0.00	0.00/33.33	0.00/16.67	

Table S7: Association between QoL Measures and Survival (OS/PFS): Univariate Cox Regression Models (Time-Dependent)

**Association between QoL Measures and Overall Survival (OS)**		
**QoL Measure**	**Hazard Ratio (95% CI)**	**P-Value**
Global Health Status	0.976 (0.943 – 1.011)	0.1796
Physical Functioning	0.957 (0.915 – 1.000	0.0504
Role Functioning	0.946 (0.898 – 0.997)	0.0369
Emotional Functioning	0.969 (0.931 – 1.009)	0.1281
Cognitive Functioning	0.991 (0.928 – 1.059)	0.7976
Social Functioning	0.967 (0.933 – 1.001)	0.0601
Fatigue	1.034 (1.001 – 1.068)	0.0458
Nausea/Vomiting	1.000 (0.961 – 1.041)	0.9905
Pain	1.031 (0.997 – 1.067)	0.0762

Association between QoL Measures and Overall Survival (OS)		
**QoL Measure**	**Hazard Ratio (95% CI)**	**P-Value**
Dyspnea	1.034 (1.000 – 1.069)	0.0484
Insomnia	1.014 (0.987 – 1.041)	0.3157
Appetite Loss	1.019 (0.992 – 1.047)	0.1642
Constipation	1.013 (0.974 – 1.052)	0.5238
Diarrhea	0.995 (0.965 – 1.026)	0.7555
Financial Difficulties	1.035 (0.992 – 1.079)	0.1142
**Association between QoL Measures and Progression-Free Survival (PFS)**		
**QoL Measure**	**Hazard Ratio (95% CI)**	**P-Value**
Global Health Status	0.958 (0.920 – 0.997)	0.0332
Physical Functioning	0.929 (0.863– 1.001)	0.0521
Role Functioning	0.910 (0.837 – 0.990)	0.0281
Emotional Functioning	0.945 (0.897 – 0.995)	0.0321
Cognitive Functioning	0.968 (0.908 – 1.033)	0.3266
Social Functioning	0.948 (0.905 – 0.994)	0.0258
Fatigue	1.030 (1.000 – 1.060)	0.0508
Nausea/Vomiting	1.033 (0.998 – 1.080)	0.1491
Pain	1.084 (1.014 – 1.159)	0.0185
Dyspnea	1.104 (0.992 – 1.229)	0.0697
Insomnia	1.018 (0.992 – 1.045)	0.1678
Appetite Loss	1.017 (0.991 – 1.044)	0.1928
Constipation	1.036 (0.995 – 1.078)	0.0857
Diarrhea	1.007 (0.971 – 1.044)	0.7138
Financial Difficulties	1.017 (0.984 – 1.052)	0.3229
